# A Large Language Model to Detect Negated Expressions in Radiology Reports

**DOI:** 10.1007/s10278-024-01274-9

**Published:** 2024-09-25

**Authors:** Yvonne Su, Yonatan B. Babore, Charles E. Kahn

**Affiliations:** 1 Department of Radiology, Perelman School of Medicine, University of Pennsylvania, 3400 Spruce Street, Philadelphia, 19104 PA USA; 2 Institute for Biomedical Informatics, University of Pennsylvania, Philadelphia, PA USA

**Keywords:** Large language models, Negated expression (negex) detection, Named entity recognition, Natural language processing, Radiology reports

## Abstract

Natural language processing (NLP) is crucial to extract information accurately from unstructured text to provide insights for clinical decision-making, quality improvement, and medical research. This study compared the performance of a rule-based NLP system and a medical-domain transformer-based model to detect negated concepts in radiology reports. Using a corpus of 984 de-identified radiology reports from a large U.S.-based academic health system (1000 consecutive reports, excluding 16 duplicates), the investigators compared the rule-based medspaCy system and the Clinical Assertion and Negation Classification Bidirectional Encoder Representations from Transformers (CAN-BERT) system to detect negated expressions of terms from RadLex, the Unified Medical Language System Metathesaurus, and the Radiology Gamuts Ontology. Power analysis determined a sample size of 382 terms to achieve *α* = 0.05 and *β* = 0.8 for McNemar’s test; based on an estimate of 15% negated terms, 2800 randomly selected terms were annotated manually as negated or not negated. Precision, recall, and F1 of the two models were compared using McNemar’s test. Of the 2800 terms, 387 (13.8%) were negated. For negation detection, medspaCy attained a recall of 0.795, precision of 0.356, and F1 of 0.492. CAN-BERT achieved a recall of 0.785, precision of 0.768, and F1 of 0.777. Although recall was not significantly different, CAN-BERT had significantly better precision (*χ*2 = 304.64; *p* < 0.001). The transformer-based CAN-BERT model detected negated terms in radiology reports with high precision and recall; its precision significantly exceeded that of the rule-based medspaCy system. Use of this system will improve data extraction from textual reports to support information retrieval, AI model training, and discovery of causal relationships.

## Introduction

The electronic health record (EHR) can serve as a rich source of information for clinical decision-making, quality improvement, and medical research, but much of its content is in the form of unstructured narrative (“free”) text [1]. In radiology, natural language processing (NLP) has found numerous applications [2, 3], including detection of critical results for diagnostic surveillance [4] and cohort building for epidemiological studies [5]. The current study harnesses named entity recognition (NER), an NLP technique that identifies and categorizes textual information; biomedical NER tasks include extraction of symptoms, signs, diseases, and treatments [6]. In radiology, NER models are designed to extract entities of interest, such as diseases and anatomical structures, from unstructured text [7, 8]. Numerous general NLP toolkits have been validated for clinical text, including cTAKES, MedTagger, and medspaCy [9–11]. However, NLP pipelines leveraging domain-specific ontologies, such as RadLex, often outperform general-purpose dictionaries in mining radiology reports for named entities [8]. Transformer-based models, which apply the notion of “attention” to leverage sentence context, can further improve the detection of named entities in radiology reports [7].

It is not enough to simply identify diseases, imaging findings, or anatomic features: radiology reports are rich in modifier terms that contain vital context without which report analysis would lead to incorrect or misleading conclusions. NLP researchers have been able to improve extraction of temporospatial relationships (e.g., “*acute thalamic* stroke”) and the negation of recognized entities (e.g., “*no* pleural effusion identified”) [12, 13]. Correct identification of negation is particularly important given the prevalence of negative findings in radiology reports [14]. If models mistakenly classify negated terms as positive instances, they will generate incorrect associations among terms. “Negation detection” evaluates whether the underlying text affirms or negates the presence of an identified entity. Making the negation-detection process precise is a critical step toward a reliable interpretation of EHR data in radiology [12, 14, 15]. Detection of negation based solely on syntax and regular expressions is difficult, as negation phrases can occur before the entity of interest, occur after it, or be may be confounded by pseudo-negation, double negatives, and other unusual negation styles [16]. Rule-based algorithms, such as NegEx, NegFinder, and NegHunter, have shown F1 scores, computed as the harmonic mean of precision and recall, from 0.84 to 0.89 for negation detection in narrative clinical text such as discharge summaries [15, 17, 18].

Large language models (LLMs) have demonstrated strong performance across various NLP tasks [19, 20]. Bidirectional encoders, such as BERT, RoBERTa, NegBERT, and CheXbert, have shown significantly improved negation detection over rule-based algorithms; models trained specifically on radiology reports, such as RadBERT, showed some of the most robust negation and uncertainty detection [21–26]. For example, negation detection in radiology reports achieved an F1 score of 0.932 for a rule-based approach and 0.961 for a BERT-based system [27]. The current study sought to compare the performance in a real-world clinical setting of a rule-based model and a pre-trained LLM on a collection of radiology reports without use of a conventional training dataset.

## Materials and Methods

### Data Collection

This retrospective study was HIPAA-compliant and was approved by the investigators’ Institutional Review Board. From a convenience sample of 1000 consecutive de-identified radiology reports from a large, multi-hospital U.S. academic health system, 16 duplicate reports were excluded to yield a study cohort of 984 reports. The sample cohort included 599 female patients (60.9%); mean patient age was 56.8 years. There were 357 radiographic exams (36.3%), 294 CTs (29.9%), 167 MRIs (16.9%), and 100 ultrasound exams (10.1%); the remaining 66 exams (6.7%) included fluoroscopy, mammography, nuclear medicine, and interventional radiology procedures. Of the 984 reports, 277 (28.2%) were dictated by a radiology trainee and subsequently edited and approved by an attending radiologist; the others were reported by an attending radiologist only. More than 90% of reports were based on a reporting template, but all reports contained mostly narrative (“free”) text.

The investigators applied the rule-based medspaCy model to identify names of diseases and imaging findings [11]. Three medical terminology sources served as reference vocabularies: the RadLex radiology lexicon (version 4.1), Unified Medical Language System Metathesaurus (version 2022AB), and Radiology Gamuts Ontology (version 1.0) [28–30]. In this study, a “term” indicates an occurrence in a radiology report of a named entity from one of the three terminology sources.

Using the terms identified by medspaCy, we compared the performance for detection of negated terms of the medspaCy model to that of the Clinical Assertion and Negation Classification Bidirectional Encoder Representations from Transformers (CAN-BERT) model, an LLM trained on large clinical datasets from MIMIC III and the i2b2/VA challenge [31]. To ensure patient data security, all text processing was completed using Python 3.7 20 on local computers without internet connections. The report text corpus was split into separate sentences for processing by the medspaCy model, which labeled terms as negated or not negated based on their position and the sentence structure. The report text also was split into sentences for the pre-trained CAN-BERT model, and special [CLS] and [SEP] tokens were added to the beginning and end of the sentences to generate hidden states for each token. CAN-BERT’s output was passed through a simple fully connected (classification) layer to label terms as negated or not negated. A cross-entropy loss function was calculated to measure the difference between the model predictions and the actual labels with an Adam optimizer to adjust the model parameters to minimize the loss function.

### Negation Definition and Validation

Each named entity identified within the text corpus was labeled as negated or not negated. An entity was classified as negated if the report explicitly asserted the entity was absent (for example, “There is no evidence of pneumothorax”). All other mentions of named entities, including uncertain mentions (e.g., “Cardiomegaly may be present”), queries (e.g., “Rule out gallstones”), and pseudo-negations (“No clear pneumothorax”), were categorized as not negated. Two fourth-year medical students manually annotated the reports under guidance of a board-certified radiologist with more than 30 years of experience. Annotations were performed independently; disagreements were resolved by consensus to establish the reference standard for comparison with annotations made by medspaCy and CAN-BERT.

### Statistical Analysis

Power analysis indicated that 382 negated terms would provide appropriate statistical power (*α* = 0.05, *β* = 0.8) to conduct McNemar’s test. Preliminary manual review of the reports yielded an estimate that 15% of terms were negated. Thus, one could expect that a sample of 2547 terms (382/15%) would yield the requisite number of negated terms; to assure an adequate sample, we chose to analyze 2800 terms. Therefore, from among all the terms that appeared in the report text corpus, 2800 terms were chosen at random and were annotated manually as negated or not negated.

The models’ performance was evaluated against the reference standard using McNemar’s test on R 4.0.3 (R Foundation, Vienna, Austria). Each model’s precision, recall, and F1 scores were calculated; the optimal operating point for CAN-BERT was selected as the one that maximized the true positive ratio minus the false positive ration. Bootstrap resampling was used to assess for statistical significance: each of 10,000 bootstrapped trials sampled 2800 times with replacement from the medspaCy and CAN-BERT predictions. A receiver operating characteristic (ROC) curve was calculated for both models; the areas under the curves (AUC) were compared using DeLong’s statistic.

## Results

**Fig. 1:**
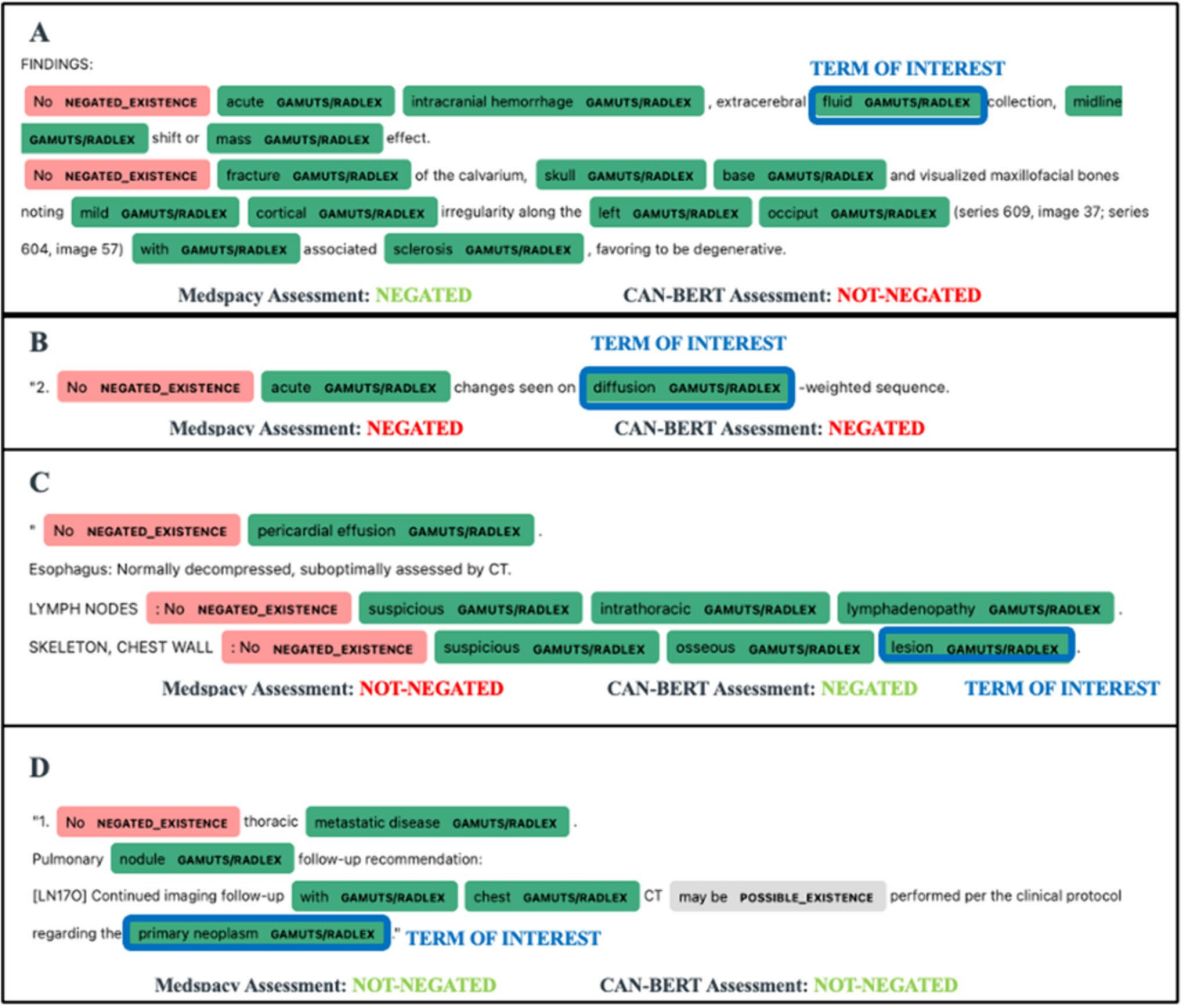
Examples of negation detection. A MedspaCy correctly identifies the term of interest as negated; CAN-BERT does not. B Both medspaCy and CAN-BERT incorrectly identified the term of interest as negated. C CAN-BERT correctly identifies the term as negated; medspaCy does not. D Both medspaCy and CAN-BERT correctly identify the term as not negated

A total of 30,210 terms were identified in the text corpus. Among the sample of 2800 terms, 387 terms (13.7%) were annotated as negated in the reference standard, which met the sample size required by the power analysis. Performance of CAN-BERT and medspaCy is shown in Fig. 1, which highlights examples of the four possible outcome scenarios for the two models. The negation status of 2053 entities was identified correctly by both models (73.3%, 2053/2800), and 82 were incorrectly identified by both models (2.9%, 82/2800). CAN-BERT correctly identified the negation status of the entities for a total of 2621 instances, whereas medspaCy did so with slightly less frequency at 2150 correct identifications (Table 1). However, medspaCy made more errors, with 650 incorrectly determined negation statuses compared to CAN-BERT’s 179 incorrect identifications. McNemar’s chi-squared test yielded a value of 304.64 (*p* < 0.001), indicating a statistically significant difference in model performance.

**Table 1:** Comparison of performance of medspaCy and CAN-BERT to correctly detect negated entities

	**medspaCy**	
**CAN-BERT**	**Correct**	**Incorrect**
Correct	2053	568
Incorrect	97	82

McNemar’s *χ*2 = 304.64

*p*-value < 0.001

**Fig. 2:**
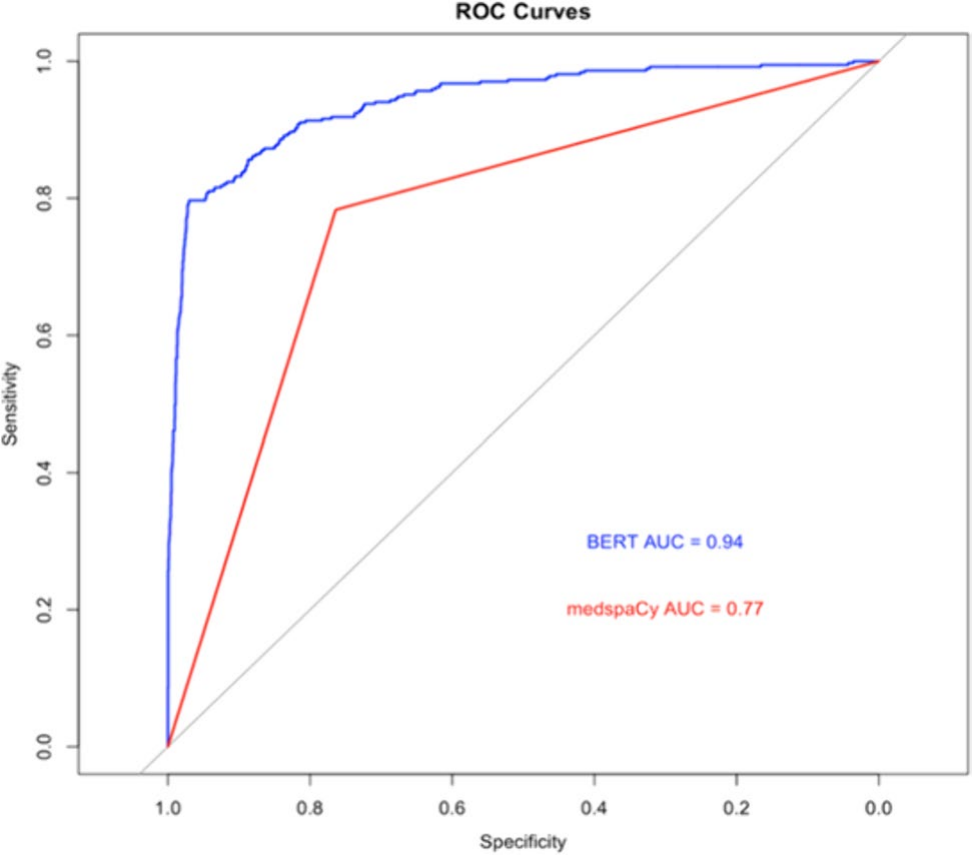
Receiver operating characteristic (ROC) curves for CAN-BERT (“BERT”) and medspaCy. AUC = area under the ROC curve

CAN-BERT outperformed medspaCy overall as shown by their receiver operating characteristic (ROC) curves (Fig. 2). The area under the ROC curve (AUC) was 0.94 for CAN-BERT and 0.77 for medspaCy; the difference was statistically significant at *p* < 0.001. The medspaCy system generated only one ROC data point because the model is rule-based. CAN-BERT’s optimal operating point yielded precision of 0.768 (vs. 0.356 for medspaCy; *p* < 0.001) and F1 score of 0.777 (vs. 0.492 for medspaCy; *p* < 0.001) (Tables 2 and 3). The models achieved similar recall.

**Table 2:** Confusion matrices for negated-term detection for (A) Medspacy and (B) CAN-BERT

**A**				
		Reference standard		
MedspaCy assertion		Negated	Not negated	Total
Negated		315	569	884
Not negated		81	1835	1916
Total		396	2404	2800

B				
	Reference standard			
CAN-BERT assertion		Negated	Not negated	Total
Negated		311	94	405
Not negated		85	2310	2395
Total		396	2404	2800

**Table 3:** Negation-detection performance of medspaCy and CAN-BERT

	**medspaCy**	**CAN-BERT**	***p-*value**
Precision	0.356	0.768	*p* < 0.001^a^
Recall	0.795	0.785	*p* = 0.727
F1 score	0.492	0.777	*p* < 0.001^a^
Specificity	0.763	0.961	*p* < 0.001^a^
Accuracy	0.768	0.936	*p* < 0.001^a^

^a^Statistically significant

## Discussion

In this study, CAN-BERT achieved better performance in negation detection than the medspaCy model. Some recent studies have suggested that LLMs have a poorer ability to detect negated expressions; these studies used common-sense knowledge to test the LLM models [32–34]. A possible explanation for this difference comes from the data each study used to test the models. In the present study, both models were tested with highly specialized textual data, namely radiology reports, and both models received specialized training in medical knowledge data from their developers.

The current investigation sought to address a critical gap in previous research: generalizability remains a significant challenge in negation detection [35]. Deep learning has been applied successfully for negation detection in limited domains, such as oncology reports, chest CT reports, and whole-body PET/CT reports [36–40]. A recent study applied negation detection to a diverse set of radiology reports, but to only 12 pathological findings based on the institution’s “list of important keywords” [41]. In contrast, the current work sought to evaluate negation detection based on comprehensive medical vocabularies to achieve a more robust and complete evaluation across a wide variety of radiology reports.

CAN-BERT’s significantly greater precision over medspaCy suggests that although rule-based systems can identify negations effectively, an LLM may have stronger performance in a novel dataset where the negation patterns are highly variable and different from the training dataset [26]. CAN-BERT’s robustness can be attributed to its training on diverse clinical datasets, which likely provided a rich linguistic foundation, enabling it to generalize better to unseen data [31]. The reliance on contextual understanding allows CAN-BERT to discern the subtleties of clinical language, often laden with jargon, abbreviations, and complex sentence structures [16].

This study has several limitations. It was based on a relatively small number of radiology reports from a single center, which may limit generalizability of the results. The text corpus may contain biases related to local choices of terminology and regional patient demographics. Furthermore, the scope of negation detection was restricted to binary classification, which, although useful for this study, may overlook the subtleties of language that convey uncertainty or gradations of certainty and potentially lead to data oversimplification. Another limitation arises from the potential for bias in the manual annotation process. Despite the oversight of experienced medical professionals, the subjective nature of interpreting medical text can introduce variability in the reference standard against which the NLP systems were evaluated. Finally, this study compared two NLP systems, and although CAN-BERT outperformed medspaCy in our analysis, it is possible that other models or combinations of models could yield different or improved results.

The outcomes of this study hold considerable significance in the context of using EHR to enhance patient care. Broadly, the ability of an NLP system to discern the presence and absence of conditions in radiology reports can streamline workflows, alleviate the workload on healthcare professionals, and potentially lead to more prompt and precise diagnoses [13, 16]. Detection of negated terms in clinical text can support research efforts to deduce causal relationships among entities in radiology reports [42]. Further research will explore the analysis of larger datasets from a variety of institutions, the classification of named entities in terms of the certainty of their presence or absence, and the development of consensus guidelines to reduce annotation bias. Additionally, further studies could evaluate the performance of a broader range of NLP systems to find the most effective tools for clinical application.

## Conclusion

Large language models such as CAN-BERT may be better suited for the complex task of detecting negated terms in complex clinical text, such as radiology reports. Subsequent research should concentrate on enhancing these models, augmenting their training datasets with a broader range of medical specializations, and formulating more complex models to interpret the full spectrum of clinical documentation. Furthermore, the results of this study could help researchers select suitable NLP tools for specific analytical tasks. Improved detection of negated expressions in radiology reports will enable more accurate text mining for information retrieval, quality improvement, automated text understanding, and training of image-based AI models [43].
